# Rare giant bilateral calvarial hyperostosis across the superior sagittal sinus secondary to brain meningioma: A case report

**DOI:** 10.3892/ol.2014.2074

**Published:** 2014-04-16

**Authors:** HENGZHU ZHANG, NAN ZHANG, LUN DONG, LEI SHE, XIAODONG WANG, ENXI XU, ZHENGCUN YAN, XIAN ZHANG

**Affiliations:** Department of Neurosurgery, Clinical Medical College, Yangzhou University, Yangzhou, Jiangsu 225001, P.R. China

**Keywords:** hyperostosis, parasagittal meningioma, tumor invasion

## Abstract

The current study presents a case of a 43-year-old female with giant bilateral calvarial hyperostosis across the superior sagittal sinus, secondary to brain meningioma. The patient presented with a huge mass in the bilateral calvarial region, and diagnoses of huge skull hyperplasia and meningioma were strongly suggested by computed tomography and magnetic resonance imaging examination. In addition, digital subtraction angiography demonstrated that the left middle meningeal artery and branches of the left superficial temporal artery were the major sources of blood supply to the tumor, with the little involvement of the right middle meningeal artery and branches of the right superficial temporal artery. The patient successfully underwent simultaneous embolization of the tumor-supplying vessels, total resection of the giant calvarial hyperostosis and intracranial tumor and skull cranioplasty. Additionally, histological study of the mass revealed a meningioma. The management of such a case presents a surgical challenge, however, the current study provides a good reference for the future treatment of similar diseases.

## Introduction

Meningioma is the most common type of benign intracranial tumor and accounts for 20–25% of all central nervous system neoplasms. Meningioma usually grows slowly and is frequently found to compress the adjacent anatomical structures, which subsequently leads to the onset of neurological symptoms and signs. Skull hyperostosis is a well-known sign of meningioma and is observed in 4.5% of all types (1). Although bone invasion and hyperostosis are common phenomena in patients with intracranial meningiomas, the basic pathomechanism is not fully understood and tumor invasion appears to be generally accepted. The current study presents a rare case of giant bilateral calvarial hyperostosis across the superior sagittal sinus, secondary to brain meningioma in a 43-year-old female. The patient successfully underwent embolization of the tumor-supplying vessels, total resection of the giant calvarial hyperostosis and intracranial tumor and skull cranioplasty in one surgical procedure. However, the management of such a case presents a surgical challenge. The patient provided written informed consent.

## Case report

In July 2011, a 43-year-old female was admitted to the Department of Neurosurgery, Clinical Medical College, Yangzhou University (Yangzhou, China) due to the progressive enlargement of a left frontoparietal mass for >30 years, with recurrent headache and hyperspasmia. The patient underwent skull tumor resection 30 years ago and the postoperative pathological diagnosis was skull hyperostosis. Following this surgery, uplift of the left frontoparietal region began gradually, which was accompanied by clinical headache and progressive enlargement of the lesion in the past two years. In addition, one week prior to admission, the patient experienced one episode of grand mal epilepsy which lasted for 15 min.

**Figure 1:**
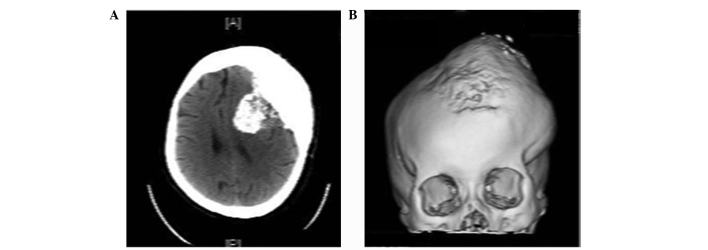
(A) Head CT virtual endoscopy scan showed a large density in the bilateral frontoparietal bone, particularly in the left side, with an unclear boundary and uneven density, measuring ~140×120 mm. (B) Head CT showed a cone-shaped intracranial meningioma below the large left frontoparietal bone density, measuring ~80×60×30 mm, which had evidently shifted the midline structure to the right. CT, computed tomography.

**Figure 2:**
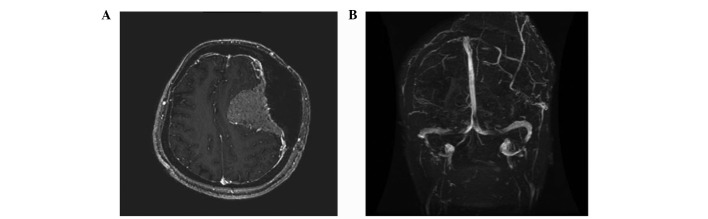
(A) Head magnetic resonance imaging revealed a bilateral frontoparietal bone hyperostosis, particularly in the left side and a cone-shaped intracranial meningioma below the bone hyperostosis. (B) Magnetic resonance angiography showed that blood to was being supplied to the lesions by the external carotid artery and that the superficial temporal artery was abnormally large. In addition, the superficial veins of the left frontoparietal lobes had been compressed downward.

**Figure 3:**
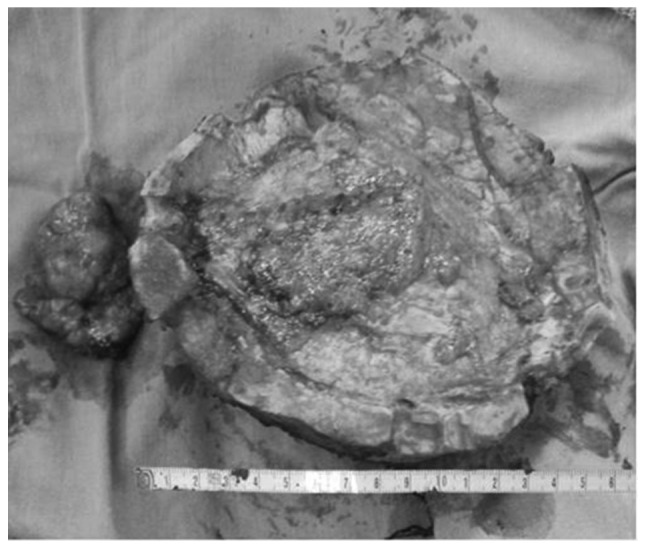
Lesions in the skull and meningioma. The meningioma volume was significantly smaller than that of the skull hyperostosis.

**Figure 4:**
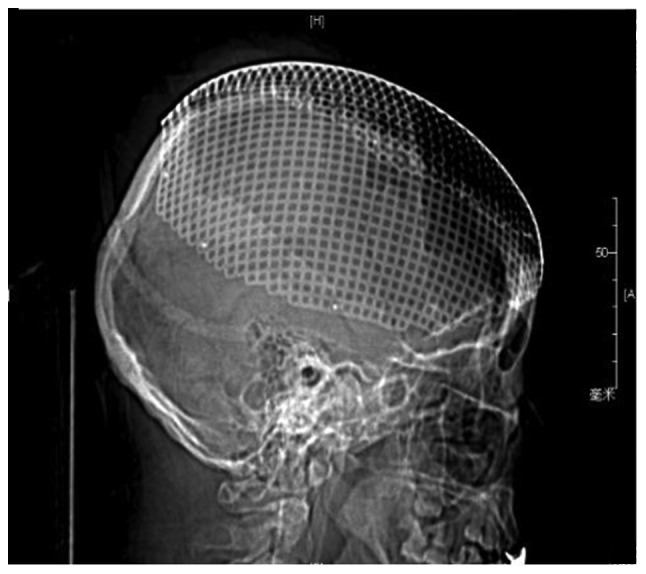
Cranioplasty with titanium mesh following the resection of the lesions.

The palpable fixed subcutaneous mass was 16×15×5 cm in size, with a hard texture, an unclear boundary and an uneven surface. The diagnoses of huge skull hyperplasia and meningioma were determined by computed tomography and magnetic resonance imaging examination (Figs. 1 and 2). In addition, digital subtraction angiography demonstrated that the left middle meningeal artery and branches of the left superficial temporal artery were the major sources of blood supply to the tumor, with little involvement of the right middle meningeal artery and branches of the right superficial temporal artery. Following embolization of the aforementioned arteries, the patient immediately underwent total resection of the giant calvarial hyperostosis and intracranial tumor, and skull cranioplasty. The tumor was removed by bilateral frontoparietal craniotomy and, during surgery, the abnormal mass, measuring 14×12×3–5 cm, exhibited a hard and uneven surface extending 3 cm from the bone surface. Based on the surgical observations, total resection of the hyperplastic skull was performed at a distance of 1 cm from the parietal eminence. The surgeons also observed that the neoplasm had invaded and destroyed the inner table of the compact bone flaps without diploe. The mass below the dura was 8×6×3 cm in size, with a soft texture and abundant blood supply. Although incomplete capsular invasion of the superior sagittal sinus was observed, the neoplasm was well-circumscribed by the cortex (Fig. 3). Following complete enucleation of the skull lesion and tumor, a cranioplasty was performed with titanium mesh (Fig. 4).

**Figure 5:**
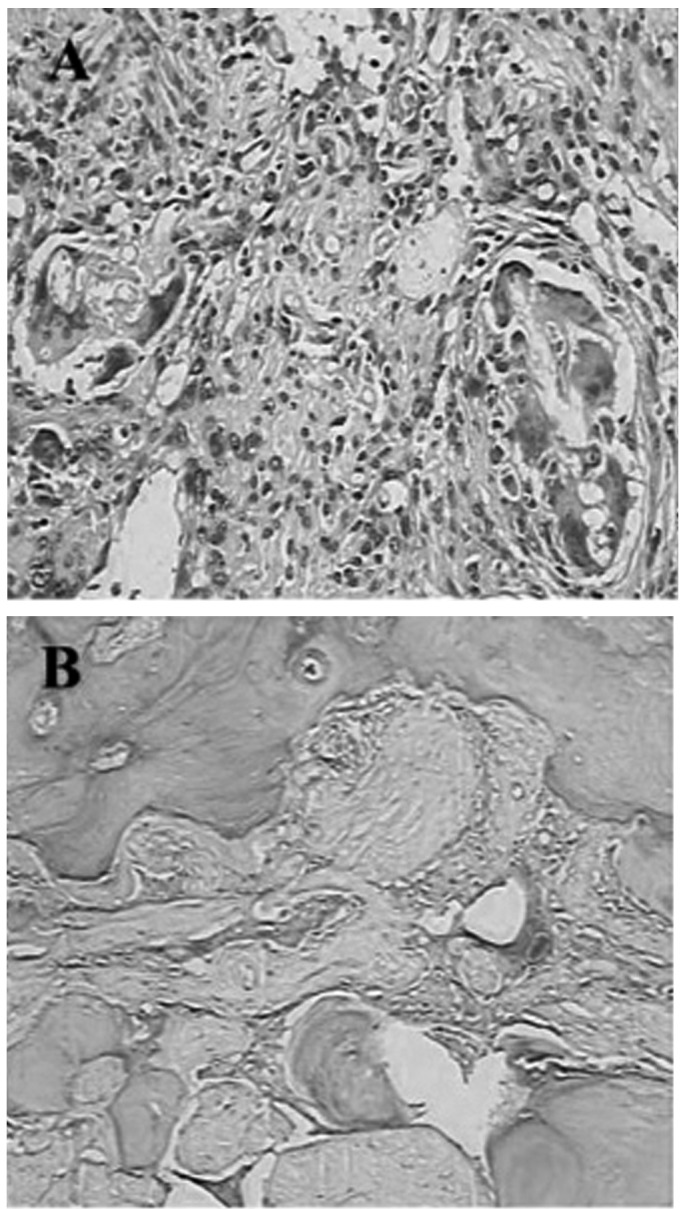
(A) Histologically, the tumor cells were oval-shaped, with hyperchromatic nuclei, cell atypia and psammoma bodies, arranged in a spiral pattern. (B) Tumor cells were revealed to have invaded all layers of the skull up to the subcutaneous tissue (stain, hematoxylin and eosin; magnification, 10×10).

The surgical specimen was routinely fixed with 10% formalin and paraffin-embedded, prior to staining with hematoxylin and eosin (magnification, 10×10). Histologically, the intracranial tumor was composed of a large number of meningothelial meningioma cells [World Health Organization (WHO) grade I] in the majority of areas and a few tumor cells exhibited severe atypism (anaplastic meningioma; WHO grade III). The external section of the tumor that involved the full thickness of the calvarial bone superiorly extended to the extracranial soft tissue. Furthermore, the gray-white tissue observed in the inner side of the examined calvarial bone was resected and measured 15×14×7 cm in size. Microscopically, the hyperostotic bone contained some meningioma tissue (Fig. 5) and the postoperative pathological diagnosis was determined as left frontotemporal meningioma accompanying calvarial bone hyperostosis. The patient was followed up for more than two years and showed no evidence of tumor recurrence.

## Discussion

The cause of associated hyperostosis in meningioma remains a point of controversy, specifically in terms of whether it presents a secondary change of the bone without tumor invasion versus direct infiltration of the bone by tumor. However, tumor invasion of the bone appears to be generally accepted, as a number of cases with hyperostosis have revealed histological tumor cell infiltration of the bone (2,3). In the present case, the tumor cells were histologically found to infiltrate from the full thickness of the skull bone to the subcutaneous tissue, which confirms the theory of tumor invasion of the bone.

However, in this case, a number of factors remain unknown, which may imply that a number of factors or an alternative pathogenesis are the cause of associated hyperostosis in meningioma. The unknown factors are as follows: The manner in which the patient’s bone hyperplasia, pathologically confirmed 30 years ago, has recurred as intracranial meningioma; whether the formation of the meningioma was caused by stimulation of the bone hyperplasia to the meningeal; or the cause of a relatively small growth of the recurrent meningioma over 30 years. Bony hyperostosis is a common sign of meningioma, in which the hyperostotic bone is usually smaller than that of the underlying tumor. However, in the present case, the sizes of hyperostotic bone and the underlying tumor were 14×12 and 8×6 cm, respectively, which contradicts the hyperostosis-associated tumor invasion theory. The following hypotheses concerning the mechanism of hyperostosis associated with meningioma may provide answers to the above questions: Preceding trauma; vascular disturbances of the bone caused by the tumor; irritation of the bone by the tumor without invasion; stimulation of osteoblast cells in the normal bone via humoral factors secreted by tumor cells; and formation of bone by the tumor itself (4). Furthermore, Pei *et al* (5) reported that the increased expression of matrix metalloproteinases (MMP)-13 and membrane-type-1-MMP in the tumor region of the hyperostosis of meningioma may contribute to the initiation of osteolysis. In addition, activated MMP-2 in hyperostotic lesions may change the physiological metabolism of the skull bone and, thus, be important for the formation of hyperostosis.

Marwah *et al* (6) termed meningioma occurring in the skull as primary intraosseous meningioma and the diagnostic criteria include the following conditions: i) Having the histological features of meningioma; ii) lesions located in the epidural or skull; and iii) no involvement of the brain tissue, arachnoid and dura. In the present case, pathological examination of the skull bone showed that the tumor cells had invaded all layers of the skull bone up to the subcutaneous tissue. Surgery confirmed the tumor to be located in the subdural space with invasion of the superior sagittal sinus, which does not meet the diagnostic criteria of the intraosseous meningiomas.

The wide extent of the tumor (bilateral extensive calvarial hyperostosis with invasion of the superior sagittal sinus and associated diffuse bilateral en plaque growth with compression of the underlying brain on the two sides) posed a formidable surgical challenge. In order to reduce bleeding during resection, it is necessary to embolize the major arteries supplying blood to the tumor, including the left middle meningeal artery and branches of the left superficial temporal artery. In addition, wherever possible, resection of the entire involved bone is recommended to prevent recurrence. In the present study, the bilateral frontoparietal flaps were extremely thick bone and, therefore, the removal of the flaps would have left a huge bilateral cranial defect involving the frontoparietal regions which may have required a significant and difficult reconstruction. An additional difficulty of the procedure is that following the removal of the bone flaps and subsequent exposure of the superior sagittal sinus, there is a risk of superior sagittal rupture, which often results in a significant amount of bleeding, even when no tearing of the superior sagittal sinus has occurred. Therefore, a large number of gelatin sponges are often required to achieve a significant hemostasis effect. Goel *et al* (7) also reported a case of extracranial extension of a meningioma, whose loss of blood exceeded 2.5 liters during the surgery. Additionally, in the postoperative phase, the patient developed disseminated intravascular coagulation disorder and suffered bleeding at multiple sites, including the surgical area, and subsequently succumbed to the disease within 8 h of the surgery. In the present case, the key to success was embolization of the major arteries supplying blood to the tumor and the tenacious protection of the superior sagittal sinus during surgery.
